# Conventional QT Variability Measurement vs. Template Matching Techniques: Comparison of Performance Using Simulated and Real ECG

**DOI:** 10.1371/journal.pone.0041920

**Published:** 2012-07-30

**Authors:** Mathias Baumert, Vito Starc, Alberto Porta

**Affiliations:** 1 School of Electrical and Electronic Engineering, The University of Adelaide, Adelaide, Australia; 2 Centre for Heart Rhythm Disorders, The University of Adelaide, Adelaide, Australia; 3 School of Medicine, The University of Ljubljana, Ljubljana, Slovenia; 4 Department of Technologies for Health, Galeazzi Orthopedic Institute, University of Milan, Milan, Italy; University Medical Center Groningen UMCG, Netherlands

## Abstract

Increased beat-to-beat variability in the QT interval (QTV) of ECG has been associated with increased risk for sudden cardiac death, but its measurement is technically challenging and currently not standardized. The aim of this study was to investigate the performance of commonly used beat-to-beat QT interval measurement algorithms. Three different methods (conventional, template stretching and template time shifting) were subjected to simulated data featuring typical ECG recording issues (broadband noise, baseline wander, amplitude modulation) and real short-term ECG of patients before and after infusion of sotalol, a QT interval prolonging drug. Among the three algorithms, the conventional algorithm was most susceptible to noise whereas the template time shifting algorithm showed superior overall performance on simulated and real ECG. None of the algorithms was able to detect increased beat-to-beat QT interval variability after sotalol infusion despite marked prolongation of the average QT interval. The QTV estimates of all three algorithms were inversely correlated with the amplitude of the T wave. In conclusion, template matching algorithms, in particular the time shifting algorithm, are recommended for beat-to-beat variability measurement of QT interval in body surface ECG. Recording noise, T wave amplitude and the beat-rejection strategy are important factors of QTV measurement and require further investigation.

## Introduction

The QT interval of body surface ECG reflects ventricular depolarization and repolarization. Prolongation of the QT interval is a clinically accepted risk factor for malignant cardiac arrhythmia and used for guiding ICD implantation and drug development [1], [2]. Measuring beat-to-beat variability in the QT interval (QTV) has received increased attention over the last 15 years, since several clinical studies provided evidence regarding the predictive value of elevated QTV causing sudden cardiac death in a variety of cardiac conditions [3], [4], [5]. Animal studies demonstrated increased QTV before the onset of drug-induced Torsades de Pointes (TdP) with a predictive value higher than that of standard QT interval assessment [6], [7], [8]. Although the mechanisms contributing to beat-to-beat QTV are incompletely understood, autonomous nervous system activity and repolarisation reserve have both been implicated [9], [10], [11], [12], in addition to the well-known action potential duration adaptation to heart rate changes [13].

While progress towards QTV analysis in clinical applications is being made [5], [14], [15], [16], [17], [18], [19], it is still constrained by insufficient formalisation of the QTV measurement process. This emphasises need for further investigations on the performance and reliability of different QT measurement algorithms [20], [21]. This is crucial as the magnitude of beat-to-beat changes in QT interval is typically of the order of few milliseconds and would considerably be affected by the accuracy of measurement. Besides issues that are related to the actual QT measurement, the separation of ‘genuine’ QTV from that solely caused by heart rate changes further complicates the clinical interpretation of QTV.

The aim of this study was to investigate the performance of template-based algorithms versus a conventional method on beat-to-beat QT interval measurement. We subjected the algorithms to simulated ECG with common signal distortions and a database of real ECG. The latter contained ECGs of patients with documented TdP, at baseline and after infusion with d,l-sotalol, a hERG-channel blocker with well established properties of action potential and QT interval prolongation as well as beta-adrenergic receptor block [22].

## Methods

### 1. Algorithms

#### 1.1 Conventional computerized QT variability measurement

Computerized measurement of the QT interval is technically challenging as evidenced by a recent ‘Computing in Cardiology’ competition [21]. Although a variety of algorithms have been proposed in the past, tangent and derivative based methods are most commonly used. Based on a previously published performance comparison for beat-to-beat QT measurement, we selected a derivative based technique as reference method [23].

The derivative based algorithm has been previously described in detail [23]. First, QRS complexes are detected based on a derivative-threshold algorithm. Parabolic fitting on the R apex is carried out to limit jitters in the R peak location. After identifying the iso-electric points before QRS, the baseline is estimated by means of cubic spline interpolation based on five cardiac beats before and after the current one and then removed. The detection of the T wave offset starts from the identification of the T wave apex, which is searched within a time window ranging between 0.15 and 0.4 times the preceding heart period. After locating the T apex, the ECG is differentiated for a constant duration that is defined by the operator on an individual basis, using a derivative finite impulse response filter, differentiating up to 25 Hz with a cut-off over 30 Hz. The T wave end is located where the absolute value of the first order derivative of the T wave down slope becomes smaller than a threshold which is proportional to the absolute value of derivative maximum. The constant of proportionality was set at 0.2. The automatic detection of the T wave end was *a posteriori* reviewed by an expert cardiologist, who validated the fiducial point identification or defined a new T wave end, using a moving calliper while watching the ECG trace. The new location was labelled as manually corrected. The QT interval was then approximated as the time distance between R apex and T wave end.

#### 1.2 Template stretching based QT variability measurement

The template stretching technique has been described in detail previously [3]. The main idea is to manually define a template QT interval by selecting the beginning of the QRS complex and the end of the T wave for one beat. The task of the algorithm is then to measure the QT interval of all other beats by determining how much each beat must be stretched or compressed in time to best match the template.

After re-sampling the original ECG to 1 kHz the location of each R wave is identified with an automated peak detection algorithm that has been proposed by Pan and Tompkins [24]. The operator then marks the start and end of the QT interval for one beat via a graphical user interface to obtain the reference QT interval. An additional marker is placed in the ST segment to define the T wave onset. The algorithm then uses this operator defined T wave template to calculate the matching error between all other T waves and the template based on the sum of squared differences. The T wave of each beat is iteratively rescaled with the aim of minimising the error function. After identifying the scaling factor that minimises the error, the product of optimum scaling factor and template T wave duration plus the constant time interval between Q onset marker and T onset marker derived from the template is calculated, providing a measure of QT interval. Baseline wander is normally removed by a 4^th^ order Butterworth high pass filter with a corner frequency of 0.3 Hz. For the purpose of this study, we discarded this pre-processing step so as to increase the comparability of algorithms.

#### 1.3 Template time shifting based QT variability measurement

The main idea of the time shifting technique is to construct separate QRS and T wave templates and shift them in time to obtain precise QT interval estimates. The algorithm is fully automated to avoid any influence of the operator and has been described in detail elsewhere [25].

First, pre-filtering is performed by a 6 pole Chebyshev low pass filter with a cut-off frequency of 125 Hz. The algorithm then detects individual beats and their P, QRS and T waves, respectively. Template beats are constructed repetitively after 60 beats to purify the template, using a signal averaging technique, in which only those beats with shapes similar to that of the template are included. When stabilized, usually one template for QRS complex and T wave, respectively, is used in the time shifting procedure. The algorithm shifts the incoming wave with respect to the template until an acceptable match is obtained, minimising the sum of squared difference. The matching of waves is performed in two sub-steps. First, a broader time interval that contains the complete wave is used to reach the best fit, where the amplitude of the incoming wave is normalized with respect to the template area under the curve. Second, the normalized wave is shifted in time to achieve the best fit in a smaller time window. For T waves only the interval between apex and end of the T wave is considered for final matching, whereas for QRS complexes the interval defined by an initial slope larger than 1/5 of the QRS amplitude is considered. To exclude premature or excessively noisy beats, the statistical behaviour of the matching error of QRS and T waves is assessed. Beats with errors outside the mean ±3 SD range for either QRS complex or T wave are rejected from analysis.

### 2. Simulated ECG

**Figure 1:**
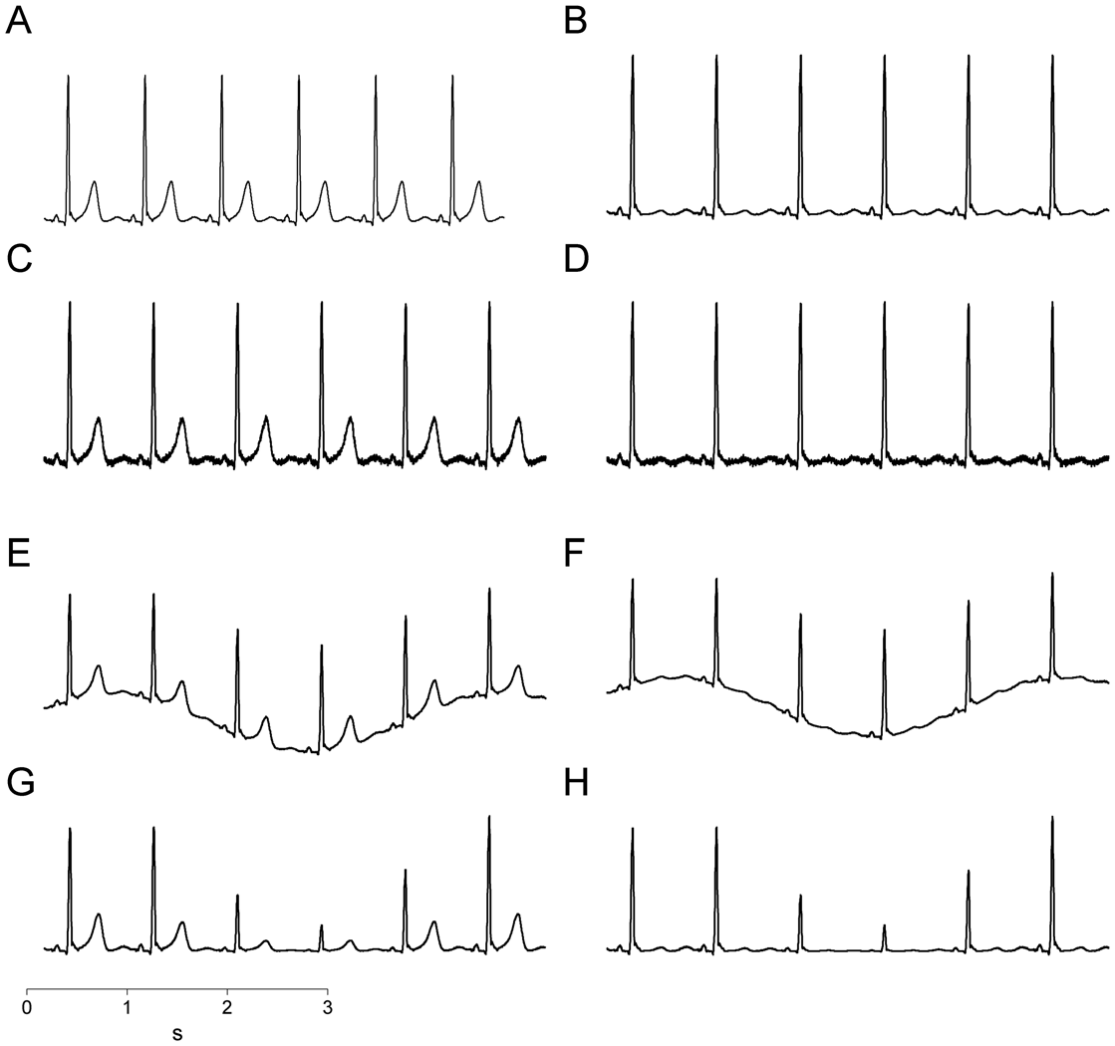
Simulations of the most common electrocardiographic artifacts. Original ECG composed of repeated identical waveforms at maximum T wave (A) and minimum T wave (B); ECG with superimposed white Gaussian noise at maximum T wave (C) and minimum T wave (D); ECG with superimposed baseline wander at maximum T wave (E) and minimum T wave (F); ECG with amplitude modulation at maximum T wave (G) and minimum T wave (H).

We derived a normal noise-free cardiac cycle (from a QRS peak to the next one) of an ECG recording obtained from a healthy young subject (age: 26 years). The original ECG was obtained from lead II and digitized using an A/D board with 12 bit resolution and a sampling rate of 1000 Hz. Given the overall range of 4096 quanta, the two R peaks spanned a range from 1983 to 2940 quanta (i.e. the R peak amplitude was 957 quanta), while the T wave spanned the range from 1984 to 2246 quanta (i.e. the T wave amplitude was 262 quanta), thus the percentage of the entire range of the A/D board occupied by the R peak and T wave was 23.4% and 6.4% respectively. The displacement of the T wave amplitude from the baseline was multiplied by *k* belonging to the set {0.1, 0.2, 0.3, 0.4, 0.5, 0.6, 0.7, 0.8, 0.9, 1.0}, thus obtaining ten cardiac beats from the original one with decreasing T wave amplitudes with *k* from 1.0 to 0.1, where *k*  = 1.0 represents the original cardiac cycle. The ten cardiac beats were then repeated 500 times, forming a set of ten synthetic signals with 500 cardiac cycles each, characterized by null variability in heart period and ventricular repolarization duration, but different T wave amplitudes (Figure 1A & B).

#### 2.1 Noisy synthetic signals

Noisy synthetic signals were obtained by adding white Gaussian noise to the original simulated signals. The mean value of the noise was zero and the standard deviation was 3% of the T wave amplitude of the original cardiac cycle (Figure 1C & D).

#### 2.2 Baseline wandering synthetic signals

Synthetic signals with baseline wander were obtained from the original simulated signals by adding a sinusoidal function with amplitude equal to that of the T wave of the original cardiac cycle and a frequency equal to 0.3 Hz (i.e. a typical human respiratory rate; Figure 1E & F).

#### 2.3 Amplitude-modulated synthetic signals

Amplitude-modulated synthetic signals were obtained by multiplying the displacement of the original simulated signals with respect to baseline by a sinusoidal function with a frequency equal to 0.3 Hz, amplitude equal to 0.7 and a mean value of one. Thus, the T wave amplitude at the apex was modulated with values ranging from 262*0.3 = 79 to 262*1.7 = 445 (Figure 1G & H).

### 3. ECG of Patients before and after D,l Sotalol Infusion

The data for this retrospective analysis were provided by the Telemetric and Holter ECG Warehouse dataset (E-OTH-12-0068-010), comprising 68 short-term 12-lead ECG recordings in patients with and without a history of drug-induced TdP. ECGs (Mortara Instrument, Milwaukee, Wisconsin) of 2–5 minute duration were recorded in the supine position at baseline and after injection of d,l-sotalol. Details of the original study protocol were published previously [26]. Briefly, patients received an intravenous sotalol perfusion over 20 minutes at a dose of 2 mg/kg with the aim of unmasking latent repolarization abnormalities. For the purpose of this study, we analysed lead II of each recording.

### 4. Statistics

QTV was quantified as standard deviation of beat-to-beat QT intervals. To compare the performance of QT measurement algorithms on simulated data we applied one-way ANOVA and the Newman-Keuls test for multiple post-hoc comparisons. To investigate the performance of QTV algorithms on real ECG before and after sotalol infusion, we applied two-way ANOVA. For a direct comparison between algorithms we computed single intra-class correlation coefficients and generated Bland-Altman plots based on absolute differences. Further, we calculated Pearson’s correlation coefficient to explore the relationship between QTV and T wave amplitude.

## Results

### 1. Comparison of QTV Measurement Techniques using Simulated ECG

#### 1.1 Effect of noise on QTV measurement accuracy

**Figure 2:**
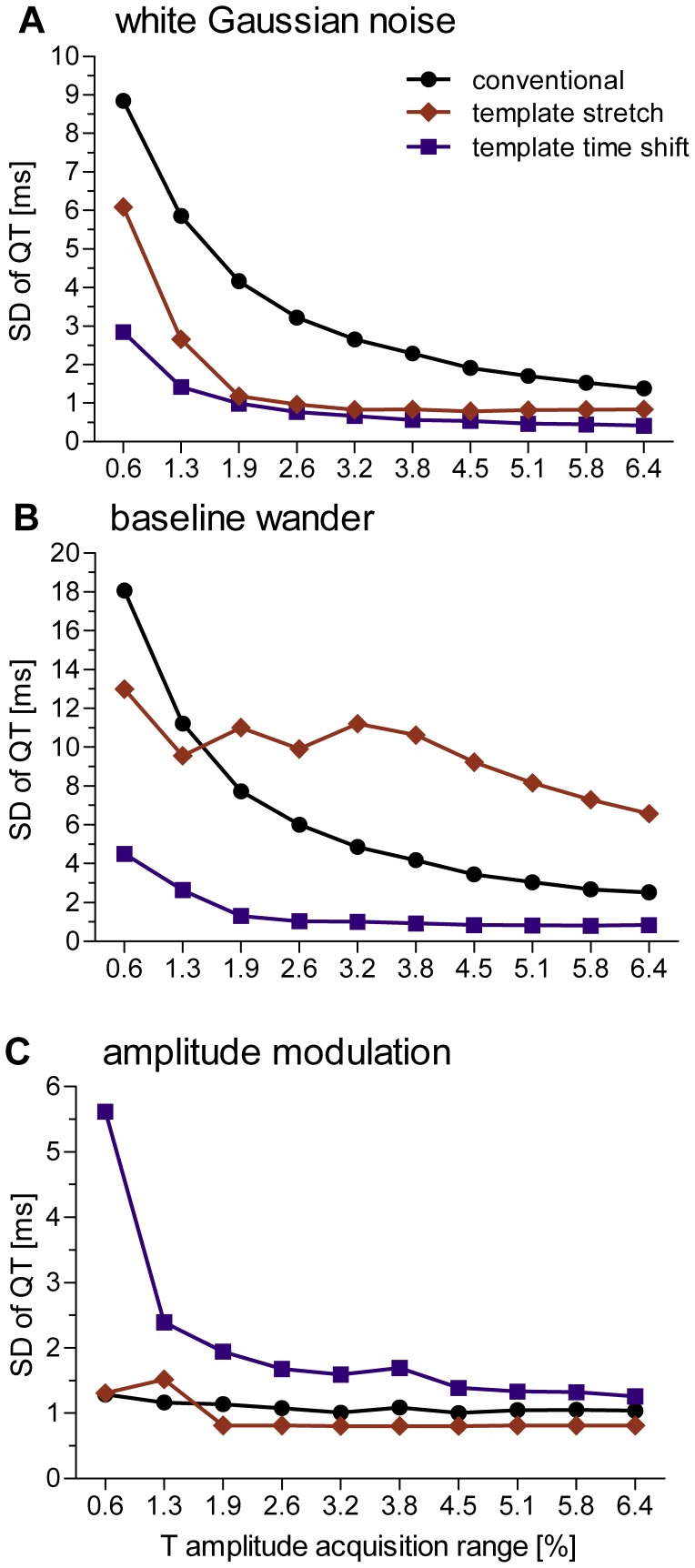
Accuracy of QT measurement algorithms. Data are expressed as standard deviation of beat-to-beat QT interval as a function of T amplitude acquisition range measured during simulated broadband noise (A), periodic baseline wander (B) and periodic amplitude modulations (C), using a conventional QT measurement algorithm (black dots), the template stretching algorithm (red diamonds) and template time shifting algorithm (blue squares).

The presence of white Gaussian noise introduced a notable amount of artificial QT variability, ranging between 1 ms at an T wave acquisition range (TWAR) of 6.4% up to 9 ms at the lowest TWAR of 0.6% (ANOVA: *p*  = 0.01). Susceptibility to noise was significantly higher when using the conventional QT measurement method compared to the template time shifting method (*p*<0.05) with intermediate values obtained using the template stretching method (see Figure 2A). Both template based methods produced errors less than 2 ms when the TWAR was greater than 1.9%. The template stretching algorithm rejected two percent of beats at the lowest TWAR and none at higher TWARs. No beat was discarded by the other two algorithms.

#### 1.2 Effect of baseline wander on QTV measurement accuracy

Baseline wander introduced artificial QT variability that ranged between one and 19 ms for TWAR values ranging between 0.6% and 6.4% (ANOVA *p*<0.001). Post-hoc comparison of algorithms showed significant differences in performance, where the template time shifting algorithm performed best and template stretching algorithm performed worst (*p*<0.05). Note that the relatively low values of QTV that were obtained with the template stretching algorithm for TWAR values below 3.8% (see Figure 2B) were caused by automated rejection of a large number of beats (88%, 74%, 60%, 43% and 10% of beats from lowest to intermediate TWAR values). No beat was discarded by the other two algorithms.

#### 1.3 Effect of amplitude modulation on QTV measurement accuracy

Amplitude modulation resulted in artificial QTV that ranged between 1 ms and 6 ms for TWAR values ranging between 0.6% and 6.4% (ANOVA *p*  = 0.008; Figure 2C). Comparing the performance of algorithms, the time shifting method introduced significantly higher artificial QTV than traditional and template stretching methods (*p*<0.05). Note that the automated beat rejection employed by the template stretching method discarded 36% of beats from each simulated recording. No beat was discarded by the other two algorithms.

### 2. Comparison of QTV Measurement Techniques using Real ECG

#### 2.1 Comparison between measurements at baseline and after sotalol infusion

**Figure 3:**
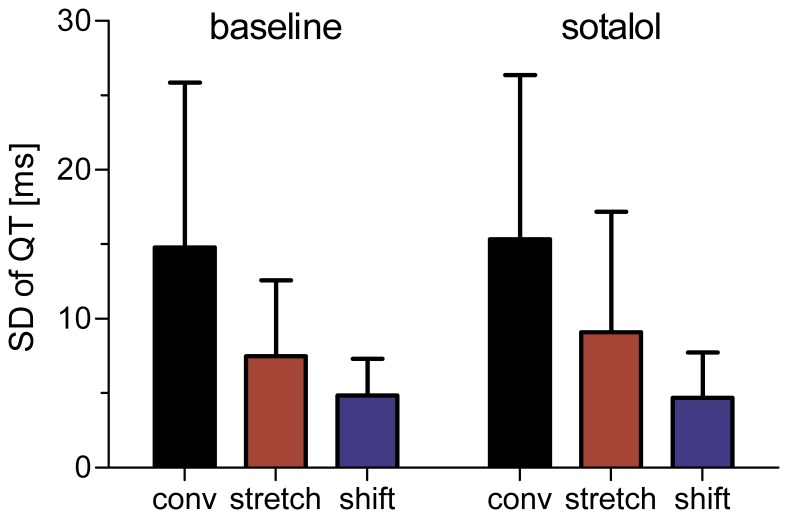
Standard deviation of beat-to-beat QT intervals in patients with reported Torsades de Pointes at baseline and after infusion of sotalol. The magnitude of measured QT variability was significantly different between algorithms. None of the algorithms detected significant changes in QTV after sotalol administration.

As expected, infusion of sotalol resulted in a significant prolongation of the rate-adjusted mean QT interval (453±47 ms vs. 518±65 ms, *p*<0.0001). Standard deviation of beat-to-beat QT intervals was not significantly affected by sotalol infusion (Figure 3). The magnitude of measured QT variability, however, was significantly different between algorithms (ANOVA: *p*<0.001). Although pair-wise post-hoc comparison did not show significant differences between algorithms, Figure 3 suggests that the conventional method measured the highest QTV values, followed by the template matching and template time shifting algorithms. Remarkably, the group average of QTV measured with the conventional method was approximately three times that of the template time shifting algorithm. Visual inspection of the error bars in Figure 3 further suggests that the template time stretching algorithm provides more consistent QTV estimates than the other two methods.

#### 2.2 Direct comparison between algorithms

**Figure 4:**
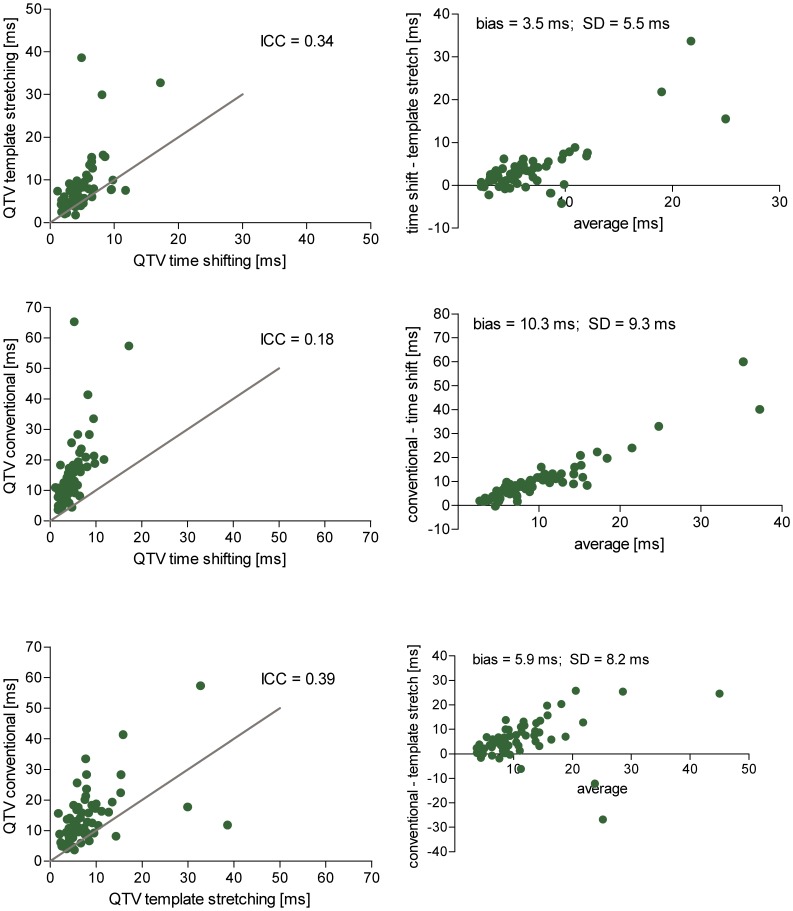
Pair-wise comparison of algorithms. Left: Scatter plots comparing QTV obtained with three different algorithms from real ECG recordings of patients before and after sotalol infusion (line of identity in grey). The overall agreement between all three algorithms (intra-class correlation coefficient) was 0.31. Right: Bland-Altman plots of absolute QTV differences observed between of algorithms.

The single intra-class correlation of QTV values measured with the three algorithms was moderate (*ICC*  = 0.31). Pair-wise comparisons of algorithms showed poorest agreement between the conventional and template time shifting algorithms, for which the single intra class correlation coefficient and the standard deviation in the Bland-Altman plots were the lowest (Figure 4). The conventional algorithm appears to measure systematically higher QTV values than the template time shifting algorithm, with intermediate values obtained with the template stretching algorithm.

#### 2.3 Correlation between QTV and T wave amplitude

**Figure 5:**
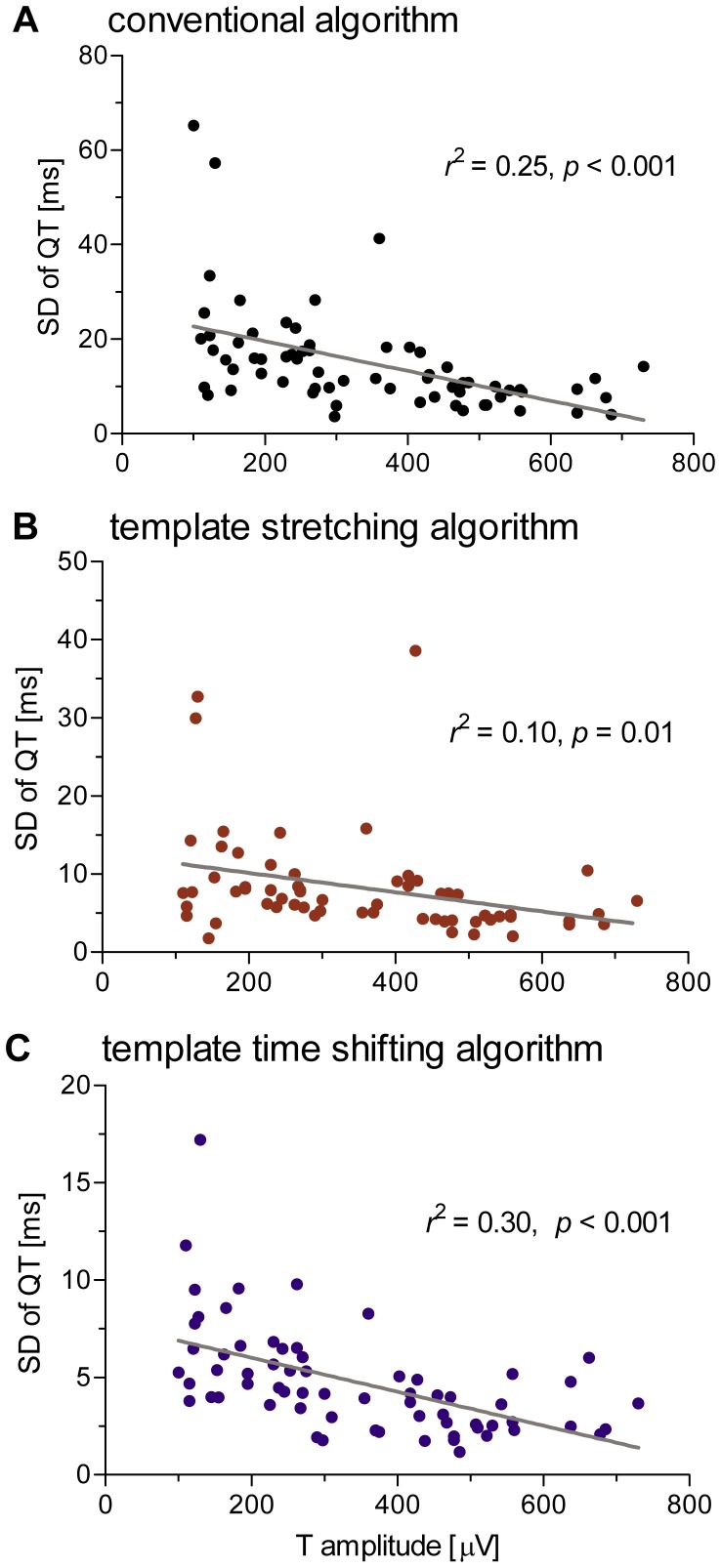
Relation between QTV and T wave amplitude. Results were obtained with the conventional (A), template stretching (B) and template time shifting (C) algorithms and show a significant negative correlation between QTV and T wave amplitude, contributing between 10% and 30% to the overall variance in QTV that was observed across patient ECGs.

Linear correlation analysis demonstrated a significant inverse relation between QTV and average T wave amplitude (Figure 5). The strength of correlation varied between algorithms and contributed between 10% and 30% to the overall variance in QTV observed in the data set (conventional: 25%; template stretching: 10%; template time shifting: 30%).

#### 2.4 Automatic beat rejection

**Figure 6:**
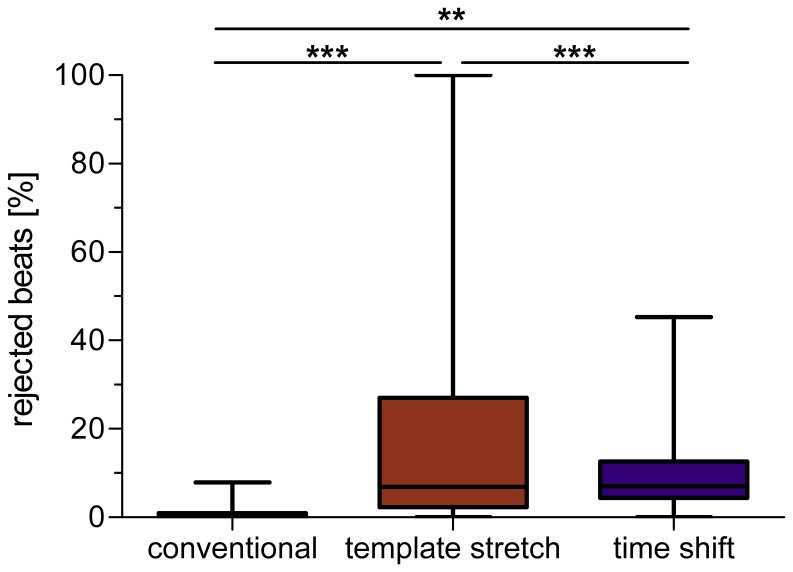
Percentage of rejected beats per record for the whole ECG data set. Note that the conventional algorithm is semi-automatic and QT intervals above a user-defined threshold were manually corrected by the operator.

The automated beat rejection that is implemented in the template stretching algorithm resulted in the exclusion of two recordings from analysis (i.e. all beats were rejected). Of the remaining 66 recordings, 17% of beats were discarded per ECG on average (see Figure 6). The automated beat rejection of the template time shifting algorithm discarded significantly fewer beats, on average ten percent per ECG. In contrast to the fully automated template based algorithms, the conventional algorithm included a post-processing stage, where the operator was able to manually correct QT intervals that exceed a user-defined threshold value (see Methods section). Manual corrections were performed on eleven percent of beats on average, which is comparable to the rejection rate of the template time shifting algorithm. After manual correction, the number of rejected beats was one per recording, on average (ANOVA *p*<0.001, all post-hoc comparisons *p*<0.01).

## Discussion

The main findings of this study are as follows: (1) the measurement technique has a significant effect on the QTV estimate; (2) there is an inverse relation between QT variability and T wave amplitude; and (3) sotalol infusion does not result in measureable QTV increase in patients with a history of TdP, despite marked QT prolongation.

### Reliability of QTV Measurement Techniques using Simulated ECG and Real ECG

Analyses based on simulated ECG suggest that the conventional QT measurement algorithm provides higher QTV estimates than template based algorithms. In particular, the conventional algorithm was more sensitive to simulated noise. The main reason for this discrepancy is that only two samples of the signal are considered by the conventional method to estimate the T wave end (i.e. the 1^st^ derivative), whereas template based algorithms consider a broader range of samples (depending on the amount of samples that form the template). These findings from simulated noisy data may also explain, at least partly, why the conventional algorithm measured systematically higher QTV in real ECG than the template algorithms. Comparing both template based algorithms, the template time shifting algorithm measured generally lower QTV than the template stretching algorithm. Although both algorithms performed similarly on simulated noisy data, the template stretching algorithm was less effective when dealing with simulated baseline wander. This disparity in performance might be partly explained by differences in template generation and matching. The template stretching algorithm considers only a single template for the whole recording, whereas the time shifting algorithm considers a set of templates. Further, the template stretching algorithm considers the whole T wave, whereas the template time shifting algorithm considers only the descending limb of the T wave for its final alignment. Thus, the time shifting algorithm has a higher overall flexibility and, thus is more robust than the template stretching algorithm. Scatter and Bland-Altman plots of real ECG suggest a better agreement between the template algorithms compared to the conventional algorithm. The good overall agreement between template algorithms, however, is blurred by outliers (see Figure 5) that were caused by the template stretching algorithm and might have originated from the less flexible template matching procedure.

### Influence of Beat Rejection on QTV Measurement

A crucial aspect in beat-to-beat QTV measurement is the strategy employed to deal with abnormal beats. Both template based algorithms follow an approach of fully automated QTV measurement and beat rejection, respectively, exploiting the error function of template matching. The underlying philosophy of the template based methods is that beat-to-beat differences in QT interval are too small to be accurately visually detected by an operator. The conventional approach, on the other hand, allows manual correction of critical beats and has the advantage of providing a time series with fewer missing beats. This may be important for studying the temporal structure of QTV, e.g. in the frequency domain [9], [27]. These different strategies for dealing with atypical beats have important implications, since irregular T waves may in fact carry the most important information on repolarization lability in view of cardiac risk stratification. This information might possibly be excluded from analysis when using fully automated template algorithms. Differences in beat rejection strategies may therefore partly explain the variation in QTV measured with the three algorithms under investigation.

### QTV after Sotalol Infusion

Despite marked QT prolongation, none of the algorithms was able to detect a significant increase in the standard deviations of QT intervals following sotalol infusion, neither in the whole group of patients (Figure 3) nor in subgroup comparisons of patients with and without a history of drug-infused TdP (data not shown). Our results are in contradiction to reports of QTV increase in dogs following sotalol administration [6], [7], [8]. This discrepancy may partly be explained by variable quality in ECG recordings. A large number of our recordings were contaminated by significant noise, which may have masked subtle increases in QTV. Our finding is in line with a previous investigation of QTV in the same data set, which did not reveal significant differences either [28]. Although it may be debatable whether to exclude noisy recordings from any QTV analysis, we decided to include all recordings to preserve a realistic setting for clinical QTV measurement. Our finding may thus emphasize an important aspect of QTV measurement in clinical routine – the requirement of high-quality noise free ECG recordings. Although we cannot exclude electrophysiological differences between animal models and patients as potential explanation for the lack of QTV changes in humans it is unlikely a main factor. Further, our investigation was limited to the standard deviation of QT intervals and we were not able to distinguish between heart rate driven QTV and genuine QTV. However, previous reports on sotalol induced QTV increase in dogs were also based on a similar approach [6], [7], [8].

In general, we may speculate that none of the proposed methods could comprehensively deal with the complexity of real ECG recordings: while the conventional method cannot deal with the large amount of broad band noise present in the recordings, thus overestimating physiological QTV, the template matching algorithms, in the attempt to limit the effect of noise, are too stiff and selective, thus resulting in an underestimation of physiological QTV. Modeling approaches that take into account the T wave morphology and its pathological changes might therefore be helpful in rejecting the large amount of noise while preserving an adequate flexibility in assessing the variability of the T wave.

All three algorithms displayed a significant inverse relationship between the T wave amplitude and QTV. This finding confirms previous observations, where inter-lead differences in T wave amplitude explained approximately 30% of QTV differences observed across the 12 standard leads [29]. Our current study of lead II ECG demonstrates that inter-individual differences in T wave amplitude are a significant contributor to QTV. The main consequences of this finding are: i) the difficulty when comparing QTV derived from different subjects/studies in absence of any indication of the T wave amplitude; ii) the necessity to account for the T wave amplitude when assessing QTV. The amplitude of the T wave should be reported to favor comparability among different studies. In the presence of baseline distortions and/or broad band noise, the amplitude of the T wave should be reported in relation to the amplitude of the baseline and/or broad band noise.

### Limitations

This study is limited to the comparison of three algorithms that were part of three different ECG analysis software programs. Differences in R wave detection and beat rejection strategies limited the comparability of actual QT measurement algorithms to some extent. We cannot distinguish between QTV introduced by either real variability of (or “jitter” in the detection of) the R or Q wave and variability of the T wave, although the former is presumably small compared to that introduced by the latter.

### Conclusions

Template matching algorithms, in particular the time shifting algorithm, are recommended for beat-to-beat variability measurement of QT interval in body surface ECG. Recording noise, T wave amplitude and beat-rejection strategies are important factors of QTV measurement and require further investigation.
